# Assessing Causal Relationship Between Human Blood Metabolites and Five Neurodegenerative Diseases With GWAS Summary Statistics

**DOI:** 10.3389/fnins.2021.680104

**Published:** 2021-12-09

**Authors:** Haimiao Chen, Jiahao Qiao, Ting Wang, Zhonghe Shao, Shuiping Huang, Ping Zeng

**Affiliations:** ^1^Department of Epidemiology and Biostatistics, School of Public Health, Xuzhou Medical University, Xuzhou, China; ^2^Center for Medical Statistics and Data Analysis, School of Public Health, Xuzhou Medical University, Xuzhou, China; ^3^Key Laboratory of Human Genetics and Environmental Medicine, Xuzhou Medical University, Xuzhou, China

**Keywords:** metabolites, neurodegenerative diseases, Mendelian randomization, metabolic pathway, amyotrophic lateral sclerosis, frontotemporal dementia, causal association

## Abstract

**Background:** Neurodegenerative diseases (NDDs) are the leading cause of disability worldwide while their metabolic pathogenesis is unclear. Genome-wide association studies (GWASs) offer an unprecedented opportunity to untangle the relationship between metabolites and NDDs.

**Methods:** By leveraging two-sample Mendelian randomization (MR) approaches and relying on GWASs summary statistics, we here explore the causal association between 486 metabolites and five NDDs including Alzheimer’s Disease (AD), amyotrophic lateral sclerosis (ALS), frontotemporal dementia (FTD), Parkinson’s disease (PD), and multiple sclerosis (MS). We validated our MR results with extensive sensitive analyses including MR-PRESSO and MR-Egger regression. We also performed linkage disequilibrium score regression (LDSC) and colocalization analyses to distinguish causal metabolite-NDD associations from genetic correlation and LD confounding of shared causal genetic variants. Finally, a metabolic pathway analysis was further conducted to identify potential metabolite pathways.

**Results:** We detected 164 metabolites which were suggestively associated with the risk of NDDs. Particularly, 2-methoxyacetaminophen sulfate substantially affected ALS (OR = 0.971, 95%CIs: 0.961 ∼ 0.982, FDR = 1.04E-4) and FTD (OR = 0.924, 95%CIs: 0.885 ∼ 0.964, FDR = 0.048), and X-11529 (OR = 1.604, 95%CIs: 1.250 ∼ 2.059, FDR = 0.048) and X-13429 (OR = 2.284, 95%CIs: 1.457 ∼ 3.581, FDR = 0.048) significantly impacted FTD. These associations were further confirmed by the weighted median and maximum likelihood methods, with MR-PRESSO and the MR-Egger regression removing the possibility of pleiotropy. We also observed that ALS or FTD can alter the metabolite levels, including ALS and FTD on 2-methoxyacetaminophen sulfate. The LDSC and colocalization analyses showed that none of the identified associations could be driven by genetic correlation or confounding by LD with common causal loci. Multiple metabolic pathways were found to be involved in NDDs, such as “urea cycle” (*P* = 0.036), “arginine biosynthesis” (*P* = 0.004) on AD and “phenylalanine, tyrosine and tryptophan biosynthesis” (*P* = 0.046) on ALS.

**Conclusion:** our study reveals robust bidirectional causal associations between servaral metabolites and neurodegenerative diseases, and provides a novel insight into metabolic mechanism for pathogenesis and therapeutic strategies of these diseases.

## Background

Neurodegenerative diseases (NDDs; Blennow et al., 2010; Parnetti, 2011), such as Alzheimer’s disease (AD), amyotrophic lateral sclerosis (ALS), frontotemporal dementia (FTD), Parkinson’s disease (PD), and multiple sclerosis (MS), are a prominent group of progressive and fatal neurological diseases currently without an effective cure, representing one of the fastest and largest increasing categories of the global disease burden especially because of aging populations (Roth et al., 2018; Bakhta et al., 2019). Therefore, identifying potential biomarkers for early diagnosis and unraveling risk factors for prevention and treatment become critical in the clinic. Although great advances have been made in discovering biomarkers and risk factors for various NDDs over the past few years (Trojanowski, 2000; Barnham et al., 2004; Emerit et al., 2004; Rachakonda et al., 2004; Amor et al., 2010; Blennow et al., 2010; Parnetti, 2011; Doty, 2017; Leng et al., 2019), the knowledge regarding the physiological and pathological mechanism underlying these diseases remains largely unclear.

As part of efforts to understand such mechanism, the relationship between metabolites and NDDs has been attracted active research attention (Wang et al., 2012; Jové et al., 2014; To et al., 2019; Chatterjee et al., 2020; Shang et al., 2020). Metabolites are the intermediate or end products that drive essential biological functions of human bodies and reflect the physiological and pathological disease phenotypes (Johnson et al., 2016; Shang et al., 2020). There is also a growing literature indicating that profiling metabolites in biofluids offers deep insights into biomarkers of NDDs (Wang et al., 2012; Mendelsohn and Larrick, 2013; Jové et al., 2014; González-Domínguez et al., 2015; Kori et al., 2016; To et al., 2019; Chatterjee et al., 2020; Shang et al., 2020). For example, it was demonstrated lipids and amino acids were associated with cognitive decline and the progression of dementia (Jiang et al., 2019); primary fatty amides in plasma were associated with brain amyloid burden and memory (Kim et al., 2019); blood metabolite changes in the periphery reflected the asymptomatic, prodromal and symptomatic stages of memory and AD (Kiddle et al., 2014; Mapstone et al., 2014). However, due to unknown confounders and reverse causality, these findings obtained from observational studies remain problematical as to whether metabolites are subsequent or consequent to NDDs, and it is also unknown whether there is a definite association between metabolites and NDDs, and whether such relationship is causal.

Recent advances in statistical genetic approaches of causal inference, along with publicly available summary statistics from large-scale genome-wide association studies (GWASs) of metabolites and NDDs provide an unprecedented opportunity to systematically evaluate their relationship through Mendelian randomization (MR; Greenland, 2000; Thomas and Conti, 2004; Tobin et al., 2004; Wheatley and Gray, 2004; Evans and Davey Smith, 2015; Zeng et al., 2019; Zeng and Zhou, 2019b; Yu et al., 2020a,b). In brief, MR is an instrumental-variable based method, which performs the causal inference with single nucleotide polymorphisms (SNPs) as instrumental variables to assess the causal effect of an exposure of focus (i.e., metabolite) on an outcome (i.e., ALS). The attractive strength of MR is that it is often less susceptible to reverse causation and confounders compared to other study designs since the two alleles of an SNP are randomly segregated under the Mendel’s law and such segregation can be considered to be independent of many unmeasured or unknown confounders. More importantly, MR can be implemented with only publicly available summary statistics of the exposure and outcome rather than individual-level genotypes and phenotypes, circumventing privacy concerns stemming from data sharing (Pasaniuc and Price, 2016). Therefore, over the past few years MR has been widely applied to disentangle the causal relationship between an exposure and an outcome in various application fields (Greenland, 2000; Thomas and Conti, 2004; Tobin et al., 2004; Wheatley and Gray, 2004; Evans and Davey Smith, 2015; Davies et al., 2018; Zeng et al., 2019; Zeng and Zhou, 2019b; Yu et al., 2020a,b).

Hereby making full use of the latest GWAS summary statistics of 486 metabolites and five major NDDs, we conducted a two-sample MR analysis to assess the causal effects of metabolites on these diseases or vice versa. Extensive sensitivity analyses, including linkage disequilibrium (LD) score regression (LDSC) and colocalization analysis (Giambartolomei et al., 2014), were also implemented to investigate whether the MR findings could be driven by genetic similarity or confounding due to LD with causal genetic loci. Overall, we found that there existed a bidirectional causal association between three metabolites and two types of NDDs (i.e., ALS and FTD). We further demonstrated that the identified associations were robust against used MR approaches and instrumental pleiotropy, and were not likely driven by shared genetic components or confounded by LD with common causal SNPs. We also identified multiple significant metabolic pathways that might be involved in the development of NDDs.

## Materials and Methods

### Genome-Wide Association Study Data Sources

We obtained summary statistics of metabolites from the metabolomics GWAS (Shin et al., 2014), which was the most comprehensive study performed to date on human blood metabolites and was a meta-analysis of two cohorts including TwinsUK and KORA F4. After quality control, a total of 486 metabolites and approximately 2.1 million SNPs up to 7,824 individuals of European ancestry were reserved for analysis (Shin et al., 2014). These metabolites can be classified as being known (309) or unknown (177). These unknown metabolites indicated that their chemical identity had not yet been conclusively established. All known metabolites can be further classified into eight broad metabolic groups (i.e., amino acid, carbohydrate, cofactors and vitamin, energy, lipid, nucleotide, peptide, and xenobiotic metabolism) (Kanehisa et al., 2012). The association of every genetic variant with individual metabolites was analyzed using a linear additive regression with age and sex as covariates.

In addition, we yielded European-only summary statistics of five NDDs (Table 1), including AD (Jansen et al., 2019), ALS (Nicolas et al., 2018), FTD (Ferrari et al., 2014), PD (Nalls et al., 2019), and MS (International Multiple Sclerosis Genetics Consortium, 2019). The Manhattan and QQ plots of *P*-values are shown in Supplementary Figures 1, 2. Although a marked departure is observed in the QQ plots of some diseases (i.e., λ = 1.13 for MS), the estimated λ_1000_ and the intercept obtained by LDSC (Bulik-Sullivan et al., 2015) indicate that the observed inflation is mainly due to polygenic signals rather than confounding factors such as population stratification or cryptic relatedness (Supplementary Table 1). Therefore, the genomic control for test statistics is not necessary for of these NDDs.

**TABLE 1:** Genome-wide association study data sets of neurodegenerative diseases employed in our analysis.

Disease	Sample size	Case	Control	Number of SNPs	Data source (PMID)
AD	455,258	71,880	383,378	13,144,351	Jansen (30617256)
ALS	80,610	20,806	59,804	8,563,029	AVS (29566793)
FTD	12,928	3,526	9,462	4,812,662	IFGC (24943344)
PD	1,474,097	56,306	1,417,791	15,317,976	IPDGC (31701892)
MS	68,379	32,367	36,012	7,930,010	IMSGC (30343897)

*AD, Alzheimer’s disease; ALS, amyotrophic lateral sclerosis; FTD, frontotemporal dementia; PD, Parkinson’s disease; MS, multiple sclerosis; SNP, single nucleotide polymorphism; AVS, the ALS Variant Server; IFGC, International FTD-Genomics consortium; IPDGC, International Parkinson’s Disease Genomics consortium; IMSGC, International Multiple Sclerosis Genetics consortium.*

### Selection of Instrumental Variables

We generated a set of uncorrelated index SNPs serving as instrumental variables for each of metabolites using the clumping procedure of PLINK (version v1.90b3.38) (Purcell et al., 2007). Specifically, we set both the primary significance level and the secondary significance level for index SNPs to be 1.00E-5, the LD, and the physical distance to be 0.10 and 500kb, respectively. Genotypes of 503 European individuals from the 1000 Genomes Project were applied as the reference panel during clumping (The 1000 Genomes Project Consortium, 2015). The relaxed statistical threshold of 1.00E-5 was employed here because of the relatively small sample size of the metabolite GWAS. In practical MR studies, a smaller significance threshold (e.g., 1.00E-5) was generally used to explain a larger variation for power enhancement when few SNPs were available for the exposure at the genome-wide significance level of 5.00E-8 (Sanna et al., 2019). In addition, to avoid horizontal pleiotropy in instrumental variables, we further removed index SNPs that were located within 1 Mb of disease-associated loci and that may be potentially related to a given neurodegenerative disease if selected genetic variants had a Bonferroni-adjusted *P*-value less than 0.05 for that disease. This was a conservative manner protecting against the pleiotropic impact of instruments to ensure valid causal inference in MR analysis (Ahmad et al., 2015; Larsson et al., 2017; Zeng et al., 2019; Zeng and Zhou, 2019a; Yu et al., 2020a).

### Causal Effect Estimation With Inverse-Variance-Weighted Mendelian Randomization Methods

For each index SNP that was utilized as instrumental variable in turn, we first examined whether it was strongly associated with the metabolite. To do so, we calculated the proportion of phenotypic variance of metabolite explained (PVE) by instruments using summary statistics using the approach proposed in Shim et al. (2015) and computed the *F* statistic to quantitatively measure the strength of instruments (Cragg and Donald, 1993; Burgess et al., 2017). The Cochran’s Q test was applied to examine the heterogeneity in effect sizes of instruments to determine whether fixed or random-effects IVW method would be utilized (Thompson and Sharp, 1999). We then undertook the inverse-variance-weighted (IVW) MR analysis for one metabolite at a time to estimate its causal effect on each of the five diseases (Burgess et al., 2017; Hartwig et al., 2017; Yavorska and Burgess, 2017). We declared an association to be statistically significant if the false discovery rate (FDR) <0.05 (Benjamini and Hochberg, 1995).

### Sensitivity Analyses for Identified Causal Associations

Based on the IVW MR analysis, we discovered several causal associations between metabolites and NDDs. However, these significant associations may reflect four explanations including: (1) *causality* from metabolites to NDDs, which indicates that the metabolites are risk factors related to the diseases; (2) *reverse causality* from NDDs to metabolites, which implies that the metabolites are biomarkers of the diseases; (3) *undetected horizontal pleiotropy*, which suggests the diseases and the metabolites may share common genetic foundation; and (4) *confounding* by LD among leading causal SNPs shared by metabolites and NDDs, which means that the observed associations are spurious (Supplementary Figure 3). Therefore, it is of importance to untangle the causal association from other explanations. To this aim, for each identified association we further implemented a series of sensitivity analyses: (i) the weighted median-based method (Bowden et al., 2016b) and the maximum likelihood method (Burgess et al., 2013) to evaluate the robustness of the discovered associations; (ii) the MR-Egger regression (Bowden et al., 2016a; Burgess and Thompson, 2017) to detect directional pleiotropic effects of instruments; (iii) the MR-PRESSO test to assess horizontal pleiotropy and examine potential instrumental outliers (Verbanck et al., 2018); (iv) the reverse causality analysis with one of NDDs as the exposure and the identified metabolite as the outcome; the instrumental variables of these NDDs were selected *via* the similar PLINK clumping procedure described above but with a genome-wide significance level of 5E-8 (Supplementary Material); (v) the multivariable MR analysis to examine the independent relationship between one associated metabolite and NDDs while adjusting for the effects of other associated metabolites (Burgess et al., 2015); (vi) LDSC to assess the overall genetic correlation (*r*_*g*_) using all available SNPs; (vii) the colocalization analysis to investigate whether the identified association between metabolites and NDDs was attributable to shared causal genetic variants (Giambartolomei et al., 2014; Supplementary Material).

### Metabolic Pathway Analysis

We finally conducted a metabolic pathway analysis for identified metabolites *via* MetaboAnalyst (Chong et al., 2018) and exploited the functional enrichment analysis module to search potential metabolite pathways for metabolites that might be involved in biological processes of the five NDDs analyzed here. Our metabolic pathway analysis included two datasets: 99 metabolite sets from The Small Molecule Pathway Database (SMPDB; Frolkis et al., 2010) and 84 metabolite sets from the KEGG database (Kanehisa et al., 2012). SMPDB is designed to support pathway discovery in clinical metabolomics, transcriptomics, proteomics, and systems biology, and further provides diagrams of human metabolic and metabolite signaling pathways.

## Results

### Causal Effects of the 486 Blood Metabolites on Neurodegenerative Diseases

**FIGURE 1:**
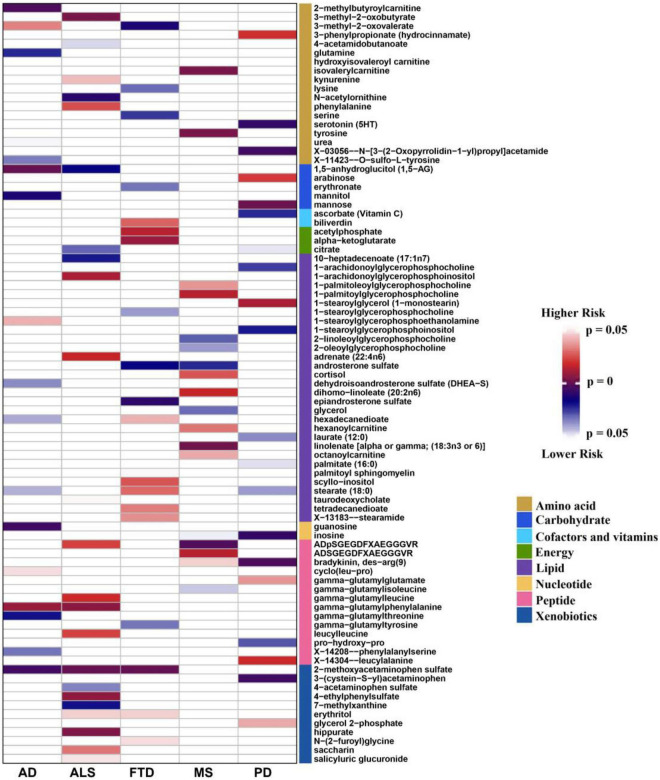
Identified causal associations between known metabolites and the risk of five neurodegenerative diseases using the IVW MR analysis. IVW, inverse-variance weighted; AD: Alzheimer’s disease; ALS: amyotrophic lateral sclerosis; FTD: frontotemporal dementia; PD: Parkinson’s disease; MS: multiple sclerosis.

The number of instrumental variables of metabolite varied from 4 to 585, with a median number of 29. These instrumental variables on average explained approximately 38.4% of phenotypic variance across all metabolites, and the minimum *F* statistic among all instruments was 20.8, indicating that weak instrumental bias is unlikely to occur and the used instruments for these metabolites were sufficiently informative (*F* statistic >10) for our MR analysis (Supplementary Table 2). Based on these instrumental variables we implemented the IVW MR analysis for each pair of metabolites and NDDs (Supplementary Table 2). We identified a total of 164 suggestive associations (136 unique metabolites) (*P* < 0.05), including 99 associations for 85 known metabolites and 65 associations for 51 unknown metabolites (Supplementary Table 3). Among these, 18, 23, 20, 20, and 18 associations of known metabolites (Figure 1) and 15, 12, 14, 13, and 11 associations of unknown metabolites (Supplementary Figure 4) are detected to be associated with the risk of AD, ALS, FTD, PD, and MS, respectively. However, after correcting multiple comparisons, we only obtain four significant associations (FDR < 0.05; involving three metabolites), including 2-methoxyacetaminophen sulfate with ALS [odd ratio (OR) = 0.971, 95% confidence intervals [CIs]: 0.961 ∼ 0.982, FDR = 1.04E-4] and FTD (OR = 0.924, 95%CIs: 0.885 ∼ 0.964, FDR = 0.048); X-11529 (OR = 1.604, 95%CIs: 1.250 ∼ 2.059, FDR = 0.048) and X-13429 (OR = 2.284, 95%CIs: 1.457 ∼ 3.581, FDR = 0.048) with FTD. Furthermore, we used a stricter *r*^2^ threshold of <0.001 in the clumping procedure to avoid the inflation of test statistics. As a result, it is shown that the four associations are still significant, with consistent effects in direction magnitude (Supplementary Table 4). 2-methoxyacetaminophen sulfate, also known as 4-(acetylamino)-3-methoxyphenyl hydrogen sulfate, is a member of the acetamide class and has a role as a drug metabolite (Mrochek et al., 1974; Bozzoni et al., 2016). Acetamides have long been recognized to be related to levels of glutathione and N-acetylcysteine, which are known biomarkers for ALS (Przedborski et al., 1996) and other NDDs such as AD (Saharan and Mandal, 2014).

It is worth noting that 2-methoxyacetaminophen sulfate also exhibits a suggestive association with AD (OR = 0.998, 95%CIs: 0.996 ∼ 0.999, *P* = 0.006), implying shared metabolic mechanisms might exist among these NDDs. Moreover, 12 metabolites are suggestively related to at least two NDDs (*P* < 0.05) (Table 2). For example, besides 2-methoxyacetaminophen sulfate, 3-methyl-2-oxovalerate is also identified to be associated with both AD and FTD. In addition, we find the direction of causal effect sizes of some metabolites is inconsistent across NDDs, including 3-methyl-2-oxovalerate on AD (OR = 1.075, 95%CIs: 1.007 ∼ 1.148, *P* = 0.031) and FTD (OR = 0.148, 95%CIs: 0.033 ∼ 0.662, *P* = 0.012), hexadecanedioate on AD (OR = 0.971, 95%CIs: 0.945 ∼ 0.999, *P* = 0.039) and FTD (OR = 1.865, 95%CIs: 1.034 ∼ 3.362, *P* = 0.038), stearate (18:0) on AD (OR = 0.932, 95%CIs: 0.872 ∼ 0.997, *P* = 0.040) and FTD (OR = 4.780, 95%CIs: 1.193 ∼ 19.150, *P* = 0.027), ADpSGEGDFXAEGGGVR on ALS (OR = 1.363, 95%CIs: 1.048 ∼ 1.773, *P* = 0.021) and MS (OR = 0.580, 95%CIs: 0.407 ∼ 0.828, *P* = 0.003), suggesting potentially different functional roles of these metabolites implicated in NDDs. In brief, these findings provide important knowledge for understanding the metabolic mechanism underlying the relationship between metabolites and NDDs.

**TABLE 2:** Overlapped known metabolites existing causal association with at least two neurodegenerative diseases.

Metabolites	AD		ALS		FTD		MS		PD	
	OR (95% CI)	*P*	OR (95% CI)	*P*	OR (95% CI)	*P*	OR (95% CI)	*P*	OR (95% CI)	*P*
M15676	1.08 (1.01 ∼ 1.15)	0.031			0.15 (0.03 ∼ 0.66)	0.012				
M20675	0.93 (0.89 ∼ 0.97)	3.2E-4	0.71 (0.53 ∼ 0.94)	0.017						
M01564			0.63 (0.41 ∼ 0.96)	0.030					0.50 (0.25 ∼ 0.99)	0.047
M31591					0.65 (0.46 ∼ 0.93)	0.017	0.86 (0.75 ∼ 0.98)	0.022		
M35678	0.97 (0.95 ∼ 1.00)	0.039			1.87 (1.03 ∼ 3.36)	0.038				
M01358	0.93 (0.87 ∼ 1.00)	0.040			4.78 (1.19 ∼ 19.2)	0.027			0.48 (0.24 ∼ 0.96)	0.037
M01123							0.84 (0.71 ∼ 1.00)	0.048	0.84 (0.73 ∼ 0.95)	0.008
M33801			1.36 (1.05 ∼ 1.77)	0.021			0.58 (0.41 ∼ 0.83)	0.003		
M34420							1.14 (1.00 ∼ 1.29)	0.043	0.84 (0.75 ∼ 0.95)	0.004
M33422	1.10 (1.03 ∼ 1.19)	0.008	1.96 (1.21 ∼ 3.17)	0.006						
M33178	0.998 (0.996 ∼ 0.999)	0.006	0.97 (0.96 ∼ 0.98)	2.3E-7	0.92 (0.89 ∼ 0.96)	2.6E-4				
M20699			1.34 (1.01 ∼ 1.79)	0.043	3.14 (1.04 ∼ 9.50)	0.043				

*M15676, 3-methyl-2-oxovalerate; M20675, 1,5-anhydroglucitol (1,5-AG); M01564, citrate; M31591, androsterone sulfate; M35678, hexadecanedioate; M01358, stearate (18:0); M01123, inosine; M33801, ADpSGEGDFXAEGGGVR; M34420, bradykinin, des-arg(9); M33422, gamma-glutamylphenylalanine; M33178, 2-methoxyacetaminophen sulfate; M20699, erythritol, AD, Alzheimer’s disease; ALS, amyotrophic lateral sclerosis; FTD, frontotemporal dementia; PD, Parkinson’s disease; MS, multiple sclerosis.*

### Results of Sensitivity Analyses

**FIGURE 2:**
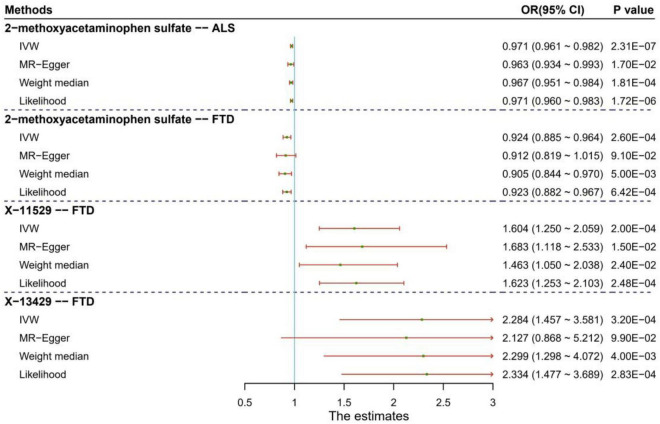
Results of sensitivity analysis for the four significant causal associations identified between human blood metabolites and two types of neurodegenerative diseases (e.g., ALS and FTD). ALS: amyotrophic lateral sclerosis; FTD: frontotemporal dementia.

To evaluate the influence of horizontal pleiotropy on our MR estimates, we conducted sensitivity and pleiotropy analyses to examine the robustness of the four associations discovered above. Here we only show some important findings, with more complete and detailed results demonstrated in the Supplementary Material. First, it is displayed that these causal associations are robust against other MR methods except the MR-Egger regression (Figure 2 and Supplementary Table 5). This might be that the MR-Egger method is substantially less efficient than other methods since it was proposed based on weaker modeling assumptions in causal inference. Second, the MR-PRESSO analysis offers little evidence for the presence of horizontal pleiotropy (*P*_MR–PRESSO_ _global_ > 0.05) and instrumental outliers (*P* > 0.05) (Supplementary Figures 5, 6). Third, the intercept of MR-Egger is not significantly deviated from zero, also indicating the absence of apparent horizontal pleiotropy (Supplementary Table 5). For the three associated metabolites identified above, to study whether the causal effect of one metabolite on FTD can be affected by the other two, we performed the multivariable MR analysis and find that, except X-11529, the direction and magnitude of the causal effects of other two metabolites (i.e., 2-methoxyacetaminophen sulfate and X-13429) are almost consistent with the unadjusted ones obtained *via* the IVW method (Supplementary Table 6), partly suggesting the independent role of these two metabolites in the risk of FTD.

We report another nine metabolites which have suggestive associations with the five NDDs (*P* < 0.05) in Table 3. For these associations, we also do not discover any evidence supporting horizontal pleiotropy, such as glutamine on AD (*P*_MR–Egger Intercept_ = 0.065, and *P*_MR–PRESSO global_ = 0.444), guanosine on AD (*P*_MR–Egger intercept_ = 0.244, and *P*_MR–PRESSO global_ = 0.739), mannose on PD (*P*_MR–Egger intercept_ = 0.260, and *P*_MR–PRESSO global_ = 0.162), citrate on PD (*P*_MR–Egger intercept_ = 0.115, and *P*_MR–PRESSO global_ = 0.408) and isovalerylcarnitine on MS (*P*_MR–Egger intercept_ = 0.222, and *P*_MR–PRESSO global_ = 0.410).

**TABLE 3:** Suggestive association metabolites passing all MR analyses at the nomial significance level of 0.05.

Metabolites	NDD	IVW		MR-Egger		Weight median		Likelihood	
		OR (95% CI)	*P*	OR (95% CI)	*P*	OR (95% CI)	*P*	OR (95% CI)	*P*
M00053	AD	0.81 (0.67 ∼ 0.97)	0.022	0.63 (0.45 ∼ 0.87)	0.010	0.72 (0.56 ∼ 0.93)	0.011	0.81 (0.67 ∼ 0.97)	0.021
M01573	AD	0.96 (0.92 ∼ 0.99)	0.006	0.91 (0.84 ∼ 0.99)	0.034	0.95 (0.91 ∼ 0.99)	0.024	0.96 (0.92 ∼ 0.99)	0.008
M00584	PD	2.69 (1.54 ∼ 4.70)	0.001	6.42 (1.19 ∼ 34.75)	0.032	3.62 (1.57 ∼ 8.39)	0.003	2.81 (1.46 ∼ 5.42)	0.002
M01564	PD	0.50 (0.25 ∼ 0.99)	0.047	0.10 (0.01 ∼ 0.85)	0.035	0.26 (0.09 ∼ 0.71)	0.009	0.49 (0.24 ∼ 0.99)	0.046
M34407	MS	1.71 (1.20 ∼ 2.45)	0.003	3.29 (1.02 ∼ 10.69)	0.047	2.53 (1.45 ∼ 4.42)	0.001	1.76 (1.11 ∼ 2.78)	0.016
M33782	ALS	1.17 (1.04 ∼ 1.31)	0.009	1.34 (1.05 ∼ 1.71)	0.023	1.21 (1.02 ∼ 1.44)	0.026	1.17 (1.02 ∼ 1.35)	0.027
M32855	FTD	2.22 (1.30 ∼ 3.78)	0.003	3.68 (1.35 ∼ 10.01)	0.014	2.72 (1.33 ∼ 5.57)	0.006	2.30 (1.34 ∼ 3.94)	0.002
M33163	FTD	9.54 (2.59 ∼ 35.09)	0.001	38.43 (1.71 ∼ 861.74)	0.024	11.68 (1.84 ∼ 74.03)	0.009	10.59 (2.76 ∼ 40.63)	0.001
M33192	PD	1.45 (1.16 ∼ 1.80)	0.001	1.74 (1.00 ∼ 3.02)	0.050	1.55 (1.16 ∼ 2.08)	0.003	1.46 (1.16 ∼ 1.83)	0.001

*M00053, glutamine; M01573, guanosine; M00584, mannose; M01564, citrate; M34407, isovalerylcarnitine; M33782, X-10346; M32855, X-11538; M33163, X-11818; M33192, X-11847, AD, Alzheimer’s disease; ALS, amyotrophic lateral sclerosis; FTD, frontotemporal dementia; PD, Parkinson’s disease; MS, multiple sclerosis.*

### Bidirectional Mendelian Randomization Examining Reverse Association From Amyotrophic Lateral Sclerosis/Frontotemporal Dementia to Metabolites

For the four significant associations between metabolites and ALS/FTD identified above, we further carried out a MR analysis using instrumental variables of ALS/FTD to estimate their reverse causal effects on metabolites (Supplementary Material). We observe that ALS/FTD also can alter the level of metabolites at the nominal significance level of 0.05 (Supplementary Table 7), such as ALS on 2-methoxyacetaminophen sulfate (OR = 0.838, 95%CIs: 0.722 ∼ 0.972, *P* = 0.020), FTD on 2-methoxyacetaminophen sulfate (OR = 1.050, 95%CIs: 1.007 ∼ 1.095, *P* = 0.021), and FTD on X-11529 (OR = 0.976, 95%CIs: 0.961 ∼ 0.991, *P* = 0.002), implying that the emergence of possible bidirectional causal relationships between these metabolites and ALS/FTD.

In this reverse MR analysis, similar results are also generated by other MR tests (*P*_Likelihood_ = 0.022 and *P*_MR–PRESSO_ = 0.038 for ALS on 2-methoxyacetaminophen sulfate; *P*_Weight–median_ = 0.030, *P*_Likelihood_ = 0.021 and *P*_MR–PRESSO_ = 3.44E-05 for FTD on 2-methoxyacetaminophen sulfate; *P*_Weight–median_ = 0.009, *P*_Likelihood_ = 0.002 and *P*_MR–PRESSO_ = 0.001 for FTD on X-11529). Moreover, there is no evidence of horizontal pleiotropy for any association (*P*_MR–Egger_ _intercept_ = 0.975 and *P*_MR–PRESSO_ _global_ = 0.558 for ALS on 2-methoxyacetaminophen sulfate; *P*_MR–Egger_ _intercept_ = 0.416 and *P*_MR–PRESSO_ _global_ = 0.999 for FTD on 2-methoxyacetaminophen sulfate; *P*_MR–Egger_ _intercept_ = 0.347 and *P*_MR–PRESSO_ _global_ = 0.918 for FTD on X-11529) (Supplementary Table 7 and Supplementary Figures 7, 8).

### Causal Association Among Identified Metabolites

**FIGURE 3:**
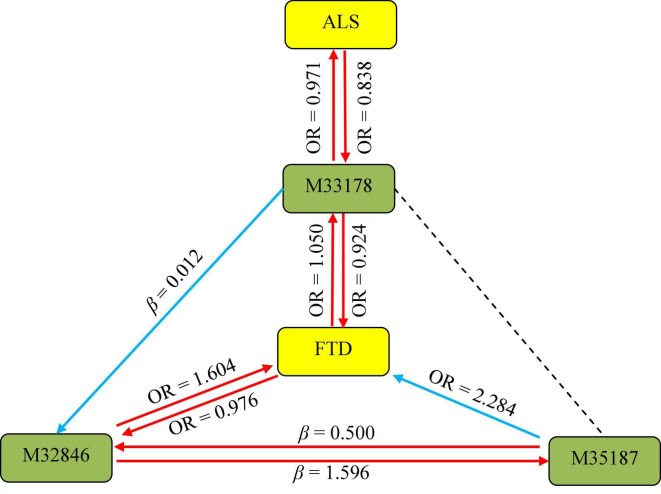
Association pathways between three metabolites and ALS/FTD with MR analysis. M33178: 2-methoxyacetaminophen sulfate; M32846: X-11529; M35187: X-13429; ALS: amyotrophic lateral sclerosis; FTD: frontotemporal dementia. The solid arrow stands for the presence of the association, while the dot line represents the absence of the association.

In order to acquire a much deeper insight into the association between the three metabolites and ALS/FTD, we performed an additional MR analysis to investigate the presence of causal relationship among these metabolites. Of interest, we ultimately observe several interaction associations between them (Supplementary Table 8), such as 2-methoxyacetaminophen sulfate on X-11529 (β = 0.012, 95%CIs: 0.002 ∼ 0.021, *P* = 0.012), X-11529 on X-13429 (β = 0.500, 95%CIs: 0.463 ∼ 0.537, *P* = 1.44E-153), suggesting that metabolites may interact to affect NDDs (Figure 3).

### Genetic Correlation and Colocalization Analyses

To examine the alternative explanation of common genetic component, we undertook the LDSC and colocalization analyses to investigate whether the genetic associations underlying metabolites and ALS/FTD were likely due to shared causal genetic variants (Supplementary Figure 3). In terms of LDSC (Supplementary Table 9), we do not observe the existence of substantial genetic correlations (*r*_*g*_ = −0.107 and *P* = 0.370 between 2-methoxyacetaminophen sulfate and ALS; *r*_*g*_ = 0.116 and *P* = 0.589 between 2-methoxyacetaminophen sulfate and FTD; *r*_*g*_ = −0.151 and *P* = 0.771 between X-11529 and FTD; and *r*_*g*_ = −0.224 and *P* = 0.675 between X-13429 and FTD). However, we cannot fully rule out the possibility of low statistical power due to small sample sizes of metabolites. Furthermore, upon performing colocalization analysis (Supplementary Table 10), we do not find any evidence of colocalization for the four associations (the posterior probability that both metabolite and NDDs are associated with common causal genetic variants < 80%), suggesting that none of these MR findings could be driven by genetic confounding by LD with causal SNPs.

### Metabolic Pathway Analysis

We were also interested in elucidating plausible metabolic pathways for the five NDDs. Therefore, we further carried out the metabolic pathway analysis using all metabolites discovered through the IVW approach (*P* < 0.05), and identified seven significant metabolic pathways for these diseases except PD (Table 4). Among them, three pathways are separately related to ALS, FTD and MS, and four are associated with AD, including “urea cycle” (*P* = 0.036), “arginine biosynthesis” (*P* = 0.004), “purine metabolism” (*P* = 0.009), and “D-glutamine and D-glutamate metabolism” (*P* = 0.042). There exists a common metabolic pathway (i.e., phenylalanine, tyrosine and tryptophan biosynthesis) shared by ALS (*P* = 0.046) and MS (*P* = 0.023).

**TABLE 4:** Significant metabolic pathways involved in the five neurodegenerative diseases.

Traits	Metabolites pathway	Involved metabolites	*P*-value	Database
AD	Urea cycle	Urea, L-Glutamine	0.0364	SMPDB
AD	Arginine biosynthesis	Urea, L-Glutamine	0.0040	KEGG
AD	Purine metabolism	Guanosine, L-Glutamine	0.0093	KEGG
AD	D-Glutamine and D-glutamate metabolism	L-Glutamine	0.0421	KEGG
ALS	Phenylalanine, tyrosine and tryptophan biosynthesis	L-Phenylalanine	0.0459	KEGG
FTD	Carnitine synthesis	L-Lysine, Oxoglutaric acid	0.0441	SMPDB
MS	Phenylalanine, tyrosine and tryptophan biosynthesis	L-Tyrosine	0.0232	KEGG

*AD, Alzheimer’s disease; ALS, amyotrophic lateral sclerosis; FTD, frontotemporal dementia; MS, multiple sclerosis; KEGG, Kyoto encyclopedia of genes and genomes; SMPDB, small molecule pathway database.*

## Discussion

Leveraging genetic variants as proxies, in the present work we assessed the causal relationship between many metabolites and five NDDs using various statistical methods (Greenland, 2000; Thomas and Conti, 2004; Tobin et al., 2004; Wheatley and Gray, 2004; Evans and Davey Smith, 2015; Davies et al., 2018; Zeng et al., 2019; Zeng and Zhou, 2019b; Yu et al., 2020a,b). Totally, we discovered 164 suggestive associations, among which four were statistically significant for three metabolites, including 2-methoxyacetaminophen sulfate (known metabolite) affecting ALS and FTD, as well as X-11529 and X-13429 (unknown metabolites) affecting FTD. Genetic studies have revealed that ALS and FTD share a high extent of common genetic origin (Wood, 2011). If 2-methoxyacetaminophen sulfate is considered a promising metabolite that has an effect on both of the two diseases, then, 2-methoxyacetaminophen sulfate can be treated as a therapeutic biomarker of diseases. Due to limited understanding of the role of this metabolite in the specific pathophysiological mechanism of ALS and FTD, this would be a suggestive finding. We further demonstrated that there also existed a reverse association between these metabolites and ALS/FTD, implying a bidirectional influence on each other. Extensive sensitivity analyses showed that these associations were robust against used MR approaches and instrumental pleiotropy, and were not likely driven by shared genetic components or confounded by LD with common causal SNPs. In addition, we found these identified metabolites interacted to influence each other, implying that there might exist a complicated network among metabolites which impact NDDs in both direct and indirect manners. It needs to highlight that a complete investigation of such role of metabolites in NDDs is beyond the scope of this work as it requires additional methodological development, which we leave for future study. Furthermore, seven significant metabolic pathways involved in the five NDDs were also detected. Our metabolic pathway analysis showed that “phenylalanine, tyrosine and tryptophan biosynthesis” was associated with both ALS and MS. It should be noted that the onset age of MS is mainly 20–40 years old (Klineova and Lublin, 2018), which is relatively younger than the average onset age of ALS. Whether phenylalanine has a mediation effect between ALS and MS needs to be further studied. In prior literature, tryptophan and competing neutral amino acid levels were found to be diminished in the plasma of patients with neurodegenerative diseases, the greatest decrease being of tryptophan (Monaco et al., 1979; Zhang et al., 2020). Evidence was also shown that higher serum phenylalanine concentrations related to immune activation are detectable in a subgroup of AD patients (Wissmann et al., 2013). The paired conversion of phenylalanine may affect not only the production of tyrosine but also the biosynthesis of the neurotransmitters dopamine, norepinephrine and epinephrine (Fernstrom and Fernstrom, 2007).

In conclusion, our findings provide an insightful perspective into the understanding of the relationship between metabolites and NDDs, and have important implications for pathology, drug development and clinical treatment. For example, given few effective drugs are available for these diseases (Al-Chalabi and Hardiman, 2013), these identified metabolites may be prioritized as candidate therapeutic targets for NDDs (Cummings et al., 2014; Lu et al., 2016), especially 2-methoxyacetaminophen sulfate for ALS or FTD. On the other hand, in terms of the findings in the reverse causality analysis, metabolites such as 2-methoxyacetaminophen sulfate can also serve as predictive biomarkers for the development of NDDs. Our study also found that some NDDs might share common metabolic mechanism, suggesting that multiple similar etiological pathways may give rise to different clinical manifestations (e.g., hexadecanedioate on AD and FTD) (Couratier et al., 2017; Ferrari et al., 2017; Broce et al., 2018; Karch et al., 2018; Abramzon et al., 2020). Overall, our study presents robust associations between multiple metabolites and NDDs, indicating the avenue for follow-up studies to improve the diagnostics, prevention and treatment of NDDs.

There are several strengths to our study. First, because it depends only on publicly available summary statistics rather than individual-level datasets, in the current work we can undertake a comprehensive MR causal inference evaluating the relationship between a large number of metabolites and NDDs in an unprecedented manner. Second, methodologically, in contrast to previous observational studies, our MR analysis implemented instrumental variable-based causal inference to assess the association between metabolites and NDDs, while minimizing the possibility of bias due to unknown confounding. Third, to provide robust MR assessment for identified associations, a wide range of sensitivity analyses were performed to distinguish causal effects from horizontal pleiotropy, reverse causation, and genetic confounding. However, this study also has some limitations. First, MR studies are generally recommended to be implemented using GWASs with large sample size; however, in our work the metabolite GWAS had a relatively small sample size, which may undermine the validity of our MR findings. Second, our study identified bidirectional causal relationships between metabolites (such as 2-methoxyacetaminophen sulfate) and NDDs (such as ALS); however, the small effect size would limit its potential utility as important biomarkers or therapeutic targets in practice. Third, we used a multivariate MR analysis to investigate whether metabolites interacted with each other to influence the causal effect of disease, but this method may be not unsuitable for unknown pleiotropy (Burgess et al., 2015). Fourth, metabolites levels are known to differ among cell and tissue types (Holmes et al., 2008; Wang et al., 2012; Shin et al., 2014); however, in this study we can only evaluate the influence of metabolites measured in blood on NDDs but unable to assess the relevance of metabolites levels in more biologically relevant tissues such as brain.

## Conclusion

In summary, our study reveals robust bidirectional causal associations between servaral metabolites and neurodegenerative diseases, and provides a novel insight into metabolic mechanism for pathogenesis and therapeutic strategies of these diseases.

## Data Availability Statement

The original contributions presented in the study are included in the article/Supplementary Material, further inquiries can be directed to the corresponding author.

## Author Contributions

PZ conceived the idea for the study. PZ, TW, SH, and HC obtained the genetic data. PZ and SH developed the study methods. PZ, TW, ZS, and HC performed the data analyses. PZ, ZS, JQ, and HC interpreted the results of the data analyses. PZ, JQ, and HC wrote the manuscript with suggestions from other authors. All authors contributed to the article and approved the submitted version.

## Conflict of Interest

The authors declare that the research was conducted in the absence of any commercial or financial relationships that could be construed as a potential conflict of interest.

## Publisher’s Note

All claims expressed in this article are solely those of the authors and do not necessarily represent those of their affiliated organizations, or those of the publisher, the editors and the reviewers. Any product that may be evaluated in this article, or claim that may be made by its manufacturer, is not guaranteed or endorsed by the publisher.

## Abbreviations

NDD: neurodegenerative disease
GWAS: genome-wide association study
MR: Mendelian randomization
AD: Alzheimer’s Disease
ALS: amyotrophic lateral sclerosis
FTD: frontotemporal dementia
PD: Parkinson’s disease
MS: multiple sclerosis
MR-PRESSO: Mendelian Randomization Pleiotropy RESidual Sum and Outlier
LDSC: linkage disequilibrium score regression
LD: linkage disequilibrium
FDR: false discover rate
SNP: single nucleotide polymorphisms
PVE: phenotypic variance explained
IVW: inverse-variance-weighted.
