# Supplementary material for: Assessing Causal Relationship Between Human Blood Metabolites and Five Neurodegenerative Diseases With GWAS Summary Statistics
Source: Front Neurosci. 2021 Dec 9;15:680104. doi: 10.3389/fnins.2021.680104 (PMC8695771; doi:10.3389/fnins.2021.680104)
Supplement: Supplementary file 2 [file Data_Sheet_1.docx]

**Supplementary Material**

## Reverse associations instrumental variable selection

In the main text, we discovered four siginificant associations (FDR < 0.05) between metabolites and NDDs, we further implemneted a reverse MR analysis with a set of instrumental variables of NDDs to estimate their causal effects on metabolites discovered. For each of NDDs we generated a set of index SNPs as instruments with the clumping procedure of PLINK (version v1.90b3.38) [[1](#_ENREF_1)]. To do so, we set the primary significance level and the secondary significance level for index SNPs to be both 5.00E-8, and the linkage disequilibrium (LD) and the physical distance to be 0.1 and 500, respectively. When clumping, the reference panel was selected with genotypes of 503 European individuals from the 1000 Genomes Project [[2](#_ENREF_2)].

To avoid horizontal pleiotropy, we further removed index SNPs of every NDD that were located within 1Mb of metabolite-associated loci and that may be potentially related to related human blood metabolites if their Bonferroni-adjusted *P* values were less than 0.05. This was a conservative way [[3](#_ENREF_3)] for protecting against the pleiotropic impact of instruments to ensure valid causal inference in MR studies [[4](#_ENREF_4), [5](#_ENREF_5)].

Ultimately, the number of instrumental variables for NDDs varied from 8 to 69 (Table S4). These instrumental variables explained 0.38~2.71% of the variance for the respective NDDs, and the minimum *F* statistic was 19.7, indicating these instrumental variables were sufficiently informative for MR analysis. According to the Cochran's Q test for instrumental heterogeneity (*P* > 0.05), we employed the fix-effects IVW method for this reverse causal analysis.

## Colocalization analysis

In order to investigate whether the significant association might be confounded by the LD between the leading SNPs for metabolites and the neurodegenerative diseases, we further performed the colocalization analysis [[6](#_ENREF_6)]. Specially, a natural question is whether two independent association signals (blood metabolites and the neurodegenerative diseases) at the same locus are consistent with a shared causal variant. We refer to this as colocalized phenotypes, and the probability that both phenotypes share a causal mechanism is greatly increased.

A key feature of the colocalization analysis is that it only requires single SNP *P* values and their MAFs, or estimated allelic effect and standard error. The SNP causality in a region of *M* genetic variants is summarized for each phenotype using an *M*-vector of 0 or 1, where 1 means that the genetic variant is causally associated with the phenotype of interest and 0 means not. Each possible pair of associations can be assigned to one of five hypotheses:

*H*_0_ (PP_0_): No association with either phenotype

*H*_1_ (PP_1_): Association with the first phenotype, not with the second

*H*_2_ (PP_2_): Association with the second phenotype, not with the first

*H*_3_ (PP_3_): Association with both phenotypes, two independent SNPs

*H*_4_ (PP_4_): Association both phenotypes, one shared SNP

Of note, PP4 offers the evidence supporting a single genetic variant affecting both phenotypes. In our colocalization analysis, we selected SNPs that were located within 100kb (±50kb) windows from each side of the instrumental variables of the four blood metabolites that were identified to be associated with ALS or FTD, and focused on a single genomic region at a time, with the interest in interpreting the pattern of LD at that locus. Broadly, in the case of either a single common causal variant or two distinct causal variants, the colocalization analysis could infer the hypotheses correctly (PP4 or PP3 > 80%). Our colocalization analysis was implemented with “coloc” package in the R (version 3.5.2) software.

## Sensitivity analysis to validate the estimated causal effect of blood metabolites on ALS and FTD

We further conducted a sensitivity and pleiotropy analysis to evaluate the robustness of the four associations between blood metabolites and ALS/FTD. It is shown that these causal relationships are robust against other MR methods; for example, *P*_Weight-median_ = 1.81E-04, *P*_MR-Egger_ = 0.017 and *P*_Likelihood_ = 1.72E-06 for 2-methoxyacetaminophen sulfate on ALS; *P*_Weight-median_ = 0.005 and *P*_Likelihood_ = 6.42E-04 for 2-methoxyacetaminophen sulfate, *P*_Weight-median_ = 0.024, *P*_MR-Egger_ = 0.015 and *P*_Likelihood_ = 2.48E-04 for X-11529, and *P*_Weight-median_ = 0.004 and *P*_Likelihood_ = 2.83E-04 for X-13429 on FTD). However, we find that not all associations are significant in the MR-Egger regression (Fig 3 and Tables S3).

In terms of the MR-PRESSO analysis, we observe no evidence of horizontal pleiotropy (*P*_MR-PRESSO Global_ = 0.087 for 2-methoxyacetaminophen sulfate on ALS; *P*_MR-PRESSO Global_ = 0.143 for 2-methoxyacetaminophen sulfate, *P*_MR-PRESSO Global_ = 0.440 for X-11529, *P*_MR-PRESSO Global_ = 0.956 for X-13429, and *P*_MR-PRESSO Global_ = 0.815 for Complement C4 on FTD), and also do not discover instrumental outliers at the nominal significance level of 0.05 (Fig S5-S6). Importantly, the intercepts of MR-Egger are also not significantly deviated from zero, indicating the absence of apparent horizontal pleiotropy (Tables S3).


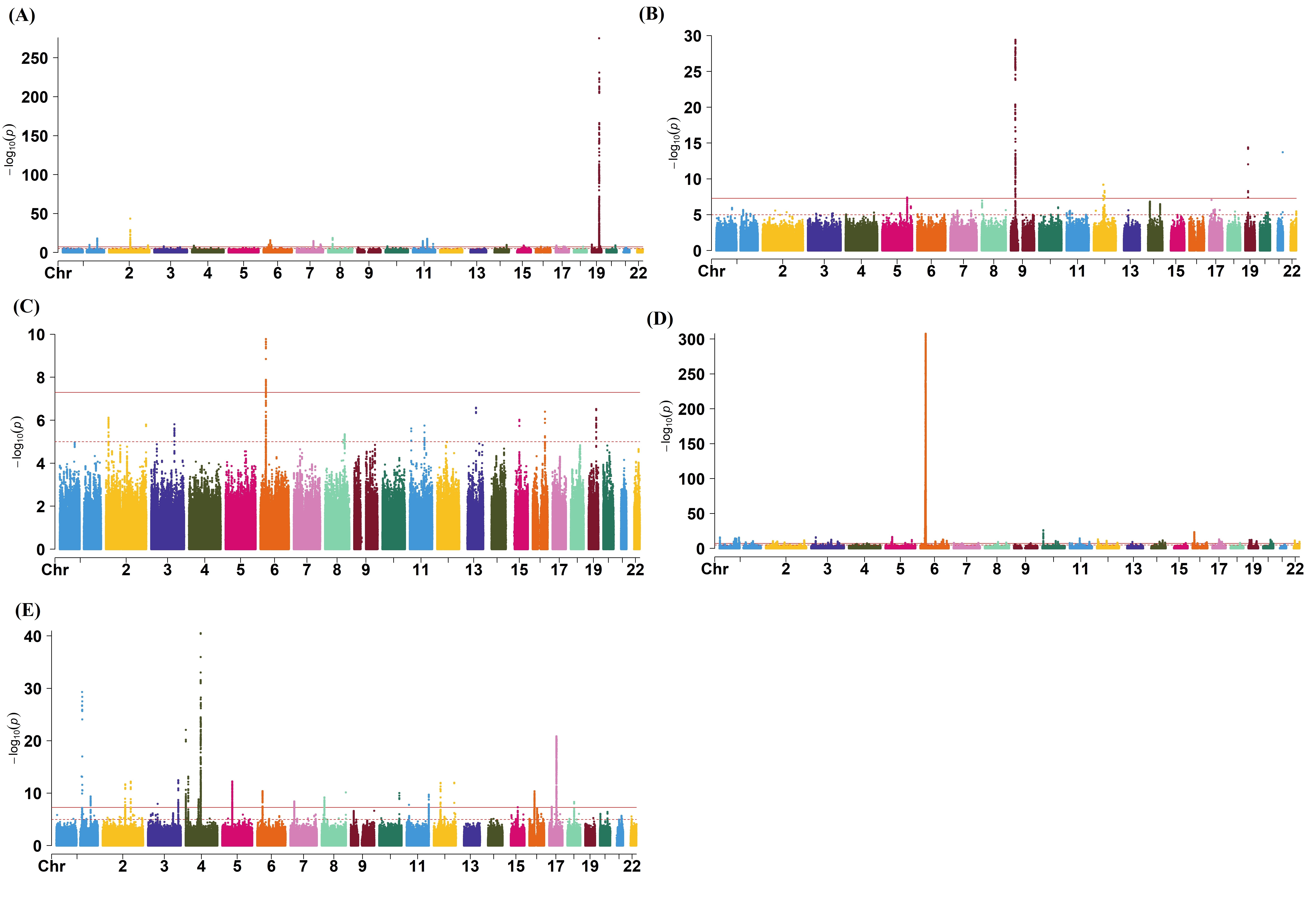


Figure S1. Manhattan plot for AD (A), ALS (B), FTD (C), MS (D), and PD (E). The two lines stand for *P* = 1.0E-05 or 5.0E-08, respectively. AD: Alzheimer’s disease; ALS: amyotrophic lateral sclerosis; FTD: frontotemporal dementia; PD: Parkinson’s disease; MS: multiple sclerosis.


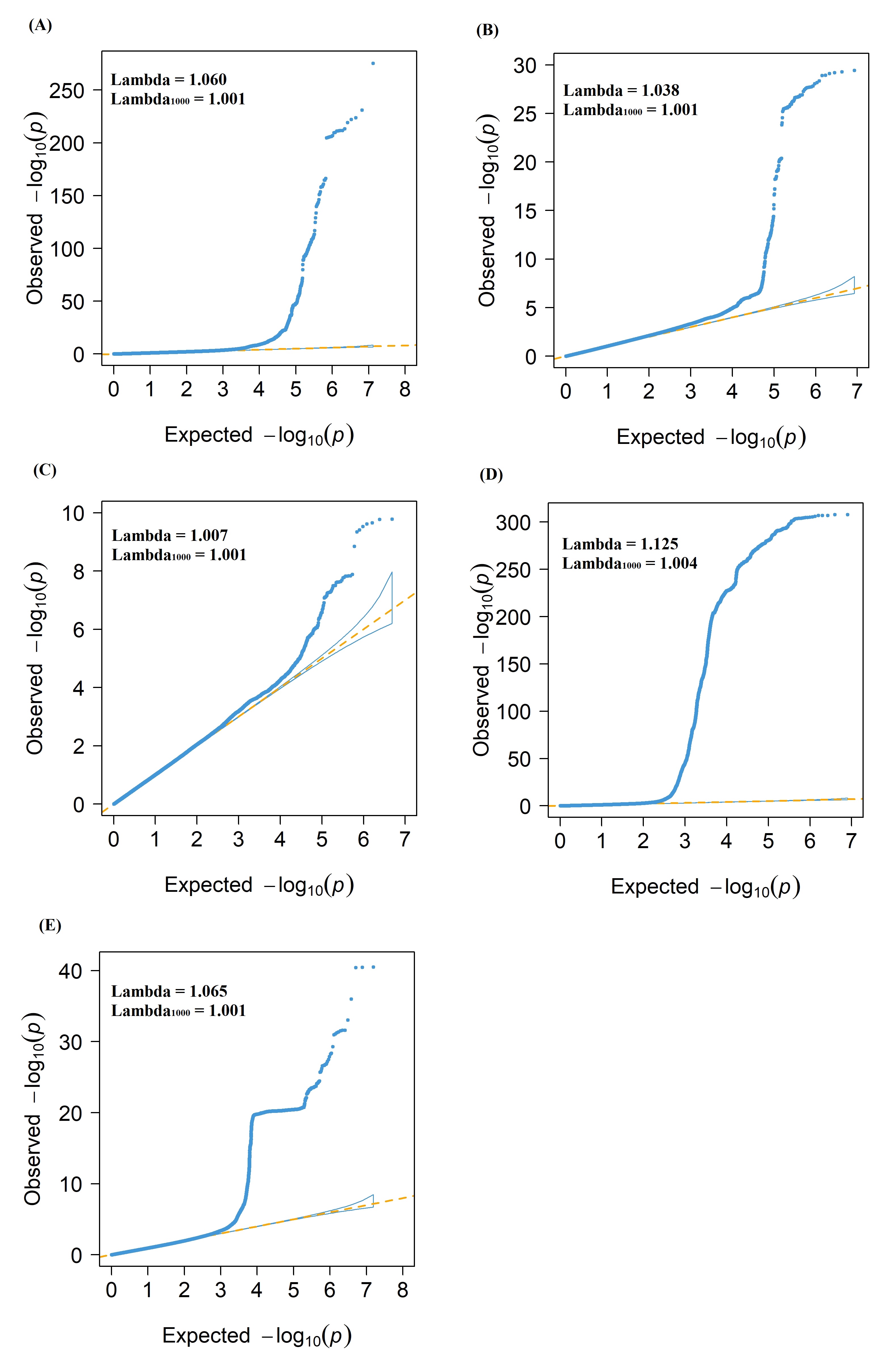


Figure S2. (A) Quantile-quantile (QQ) plot for AD (A), ALS (B), FTD (C), MS (D), and PD (E). These QQ plots display the observed meta-analysis *P* values versus the expected *P* values under the null model of no associations in the -log_10_*P* scale. The estimated λ_1000_ and the estimated LD Score regression intercept indicate that the observed inflation is mainly due to polygenic signals rather than major confounding factors including population stratification; AD: Alzheimer’s disease; ALS: amyotrophic lateral sclerosis; FTD: frontotemporal dementia; PD: Parkinson’s disease; MS: multiple sclerosis. Thus, the genomic control is not necessary.

M1: causality

valid target

variants

metabolite

NDDs

IVW + weighted median + likelihood method

M2: reverse causality

biomarker for diseases

variants

NDDs

metabolites

bidirectional MR

M3: horizontal pleiotropy

invalid target

MR-Egger + MR-PRESSO

variants

NDD

metabolite

M4: linkage disequilibrium

invalid target

variant 1

metabolite

NDD

variant 2

LD

LDSC + colocalization

Figure S3. **M1** **causality**: the cuasal associations between metabolites and neurodegenerative diseases; **M2 reverse causality**: the reverse causality between neurodegenerative diseases and metabolites; **M3 horizontal pleiotropy**: variants influence both metabolites and neurodegenerative diseases; **M4 linkage disequilibrium**: variant 1 that influences metabolites is correlated with variant 2 that influences the risk of NDDs; LDSC: linkage disequilibrium score regression; IVW: inverse variance weighted method; MR-PRESSO: Mendelian Randomization Pleiotropy RESidual Sum and Outlier test.


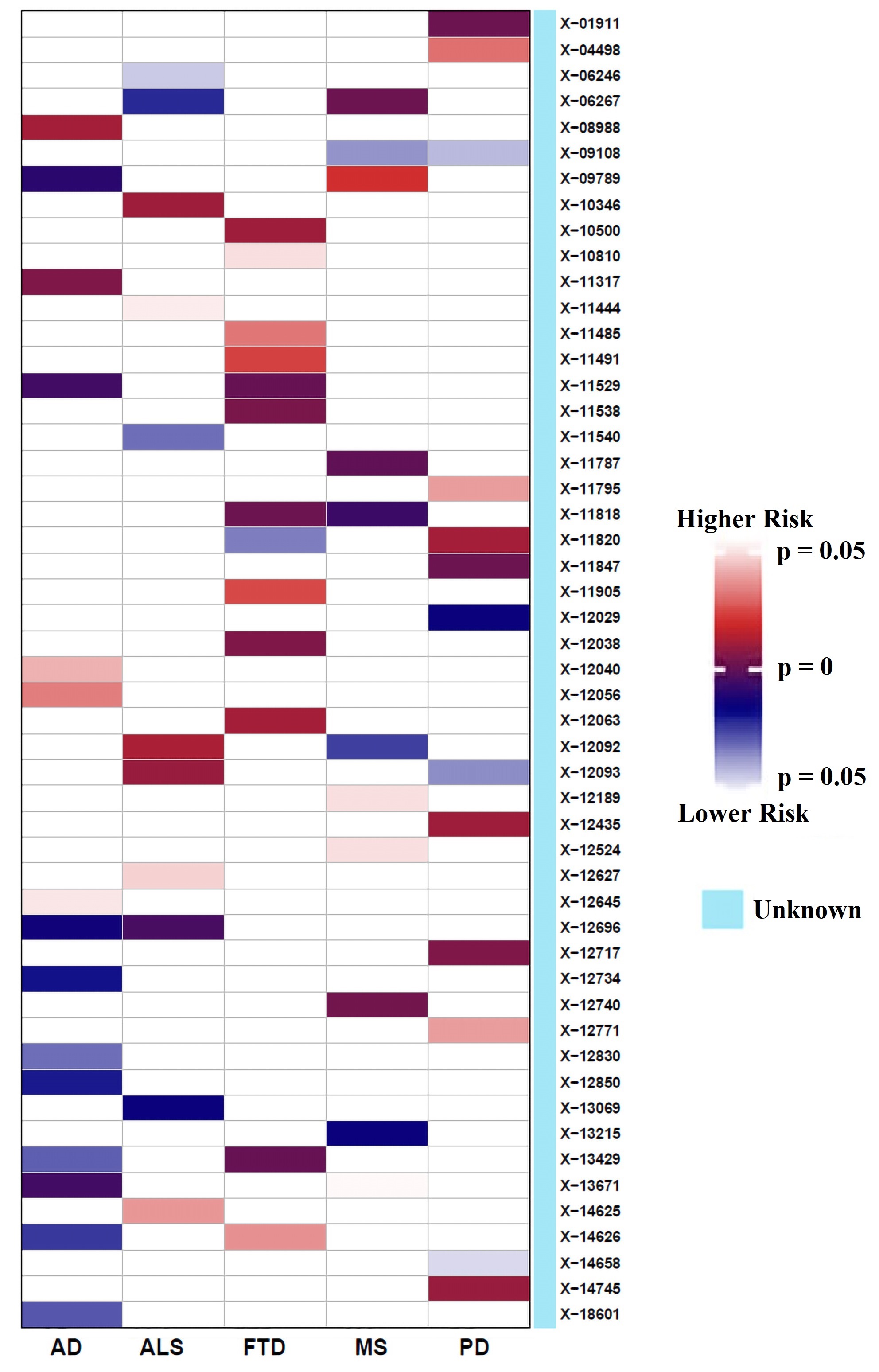


Figure S4. Identified causal associations between unknown metabolites and the risk of five neurodegenerative diseases using the IVW Mendelian randomization analysis. IVW, inverse-variance weighted; AD: Alzheimer’s disease; ALS: amyotrophic lateral sclerosis; FTD: frontotemporal dementia; PD: Parkinson’s disease; MS: multiple sclerosis.


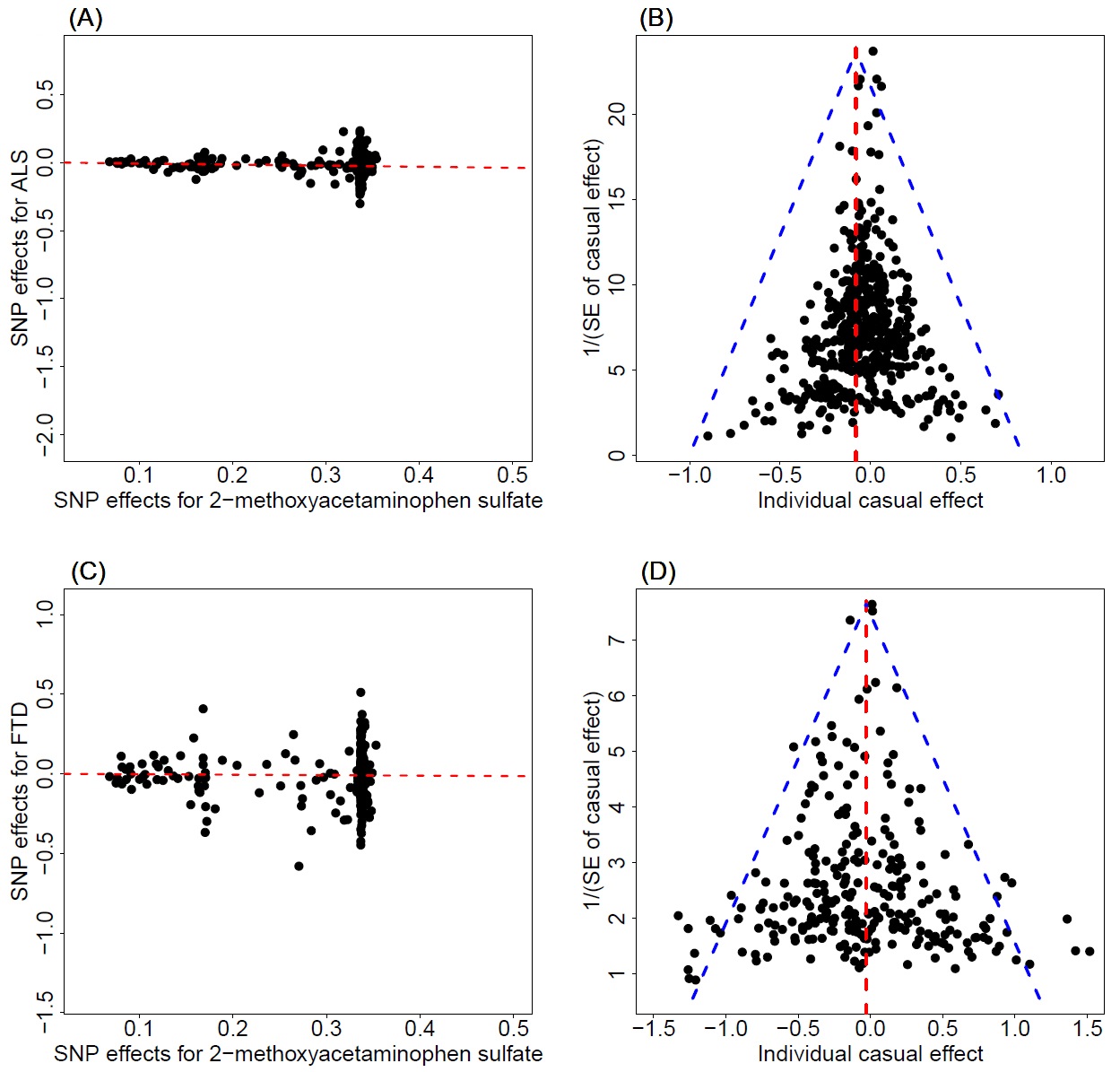


Figure S5. (A) Relationship between the SNP effect size estimates of 2-methoxyacetaminophen sulfate (x-axis) and the corresponding effect size estimates of ALS (y-axis). In the plot, the 95% confidence intervals (CIs) for the effect sizes of instruments on 2-methoxyacetaminophen sulfate are shown as horizontal lines, while the 95%CIs for the effect sizes of instruments on ALS are shown as vertical lines. The line in red represents the estimated causal effect of 2-methoxyacetaminophen sulfate on ALS with the IVW method; (B) Funnel plot for single causal effect estimates of 2-methoxyacetaminophen sulfate on ALS; the horizontal dot line denotes the overall estimated causal effect with IVW method; (C) Relationship between the SNP effect size estimates of 2-methoxyacetaminophen sulfate (x-axis) and the corresponding effect size estimates of FTD (y-axis). In the plot, the 95%CIs for the effect sizes of instruments on 2-methoxyacetaminophen sulfate are shown as horizontal lines, while the 95%CIs for the effect sizes of instruments on FTD are shown as vertical lines. The line in red represents the estimated causal effect of 2-methoxyacetaminophen sulfate on FTD with the IVW method; (D) Funnel plot for single causal effect estimates of 2-methoxyacetaminophen sulfate on FTD; the horizontal dot line denotes the overall estimated causal effect with IVW method.


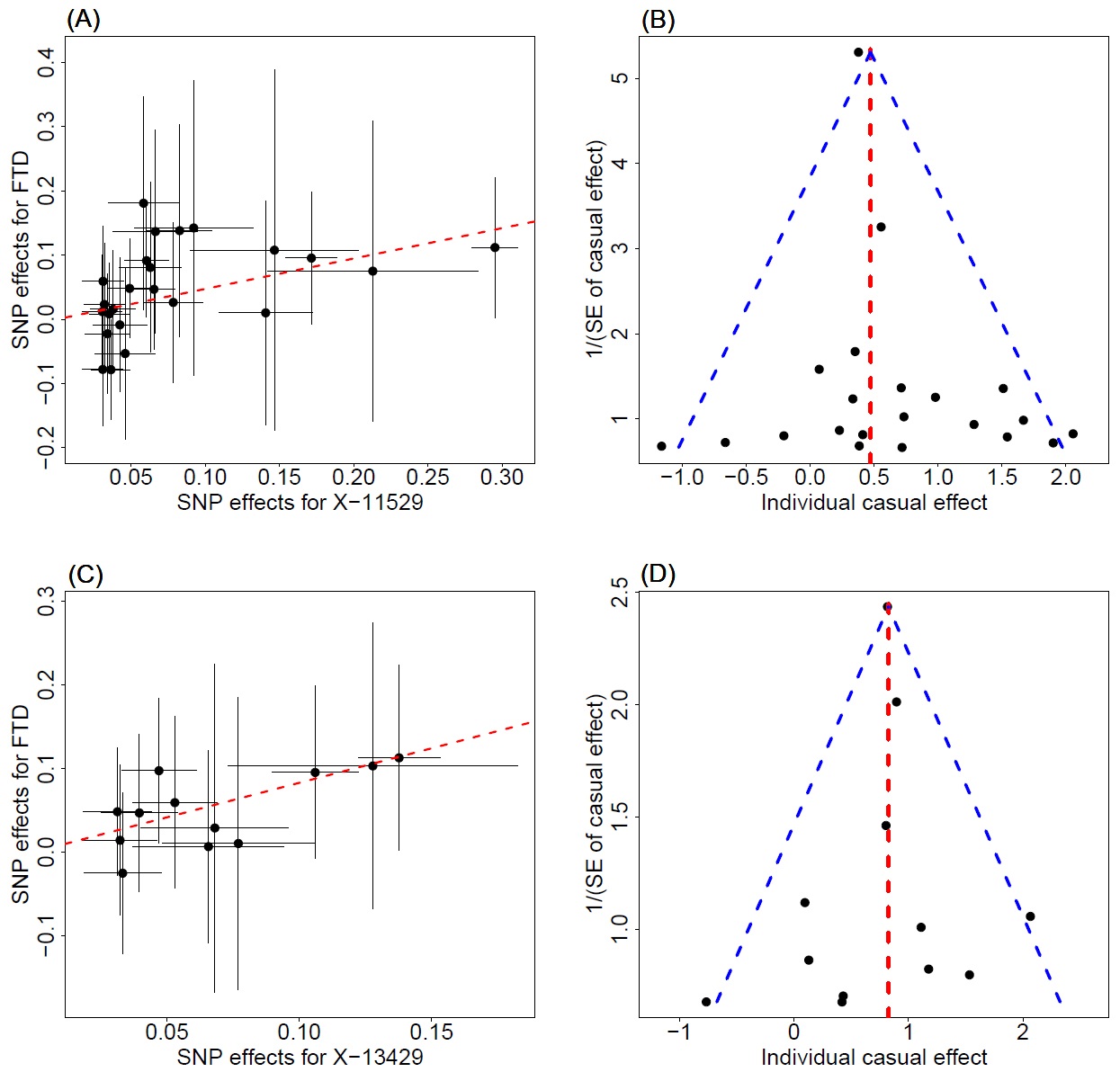


Figure S6. (A) Relationship between the SNP effect size estimates of X-11529 (x-axis) and the corresponding effect size estimates of FTD (y-axis). In the plot, the 95%CIs for the effect sizes of instruments on X-11529 are shown as horizontal lines, while the 95%CIs for the effect sizes of instruments on FTD are shown as vertical lines. The line in red represents the estimated causal effect of X-11529 on FTD with the IVW method; (B) Funnel plot for single causal effect estimates of X-11529 on FTD; the horizontal dot line denotes the overall estimated causal effect with IVW method; (C) Relationship between the SNP effect size estimates of X-13429 (x-axis) and the corresponding effect size estimates of FTD (y-axis). In the plot, the 95%CIs for the effect sizes of instruments on X-13429 are shown as horizontal lines, while the 95%CIs for the effect sizes of instruments on FTD are shown as vertical lines. The line in red represents the estimated causal effect of X-13429 on FTD with the IVW method; (D) Funnel plot for single causal effect estimates of X-13429 on FTD; the horizontal dot line denotes the overall estimated causal effect with IVW method.


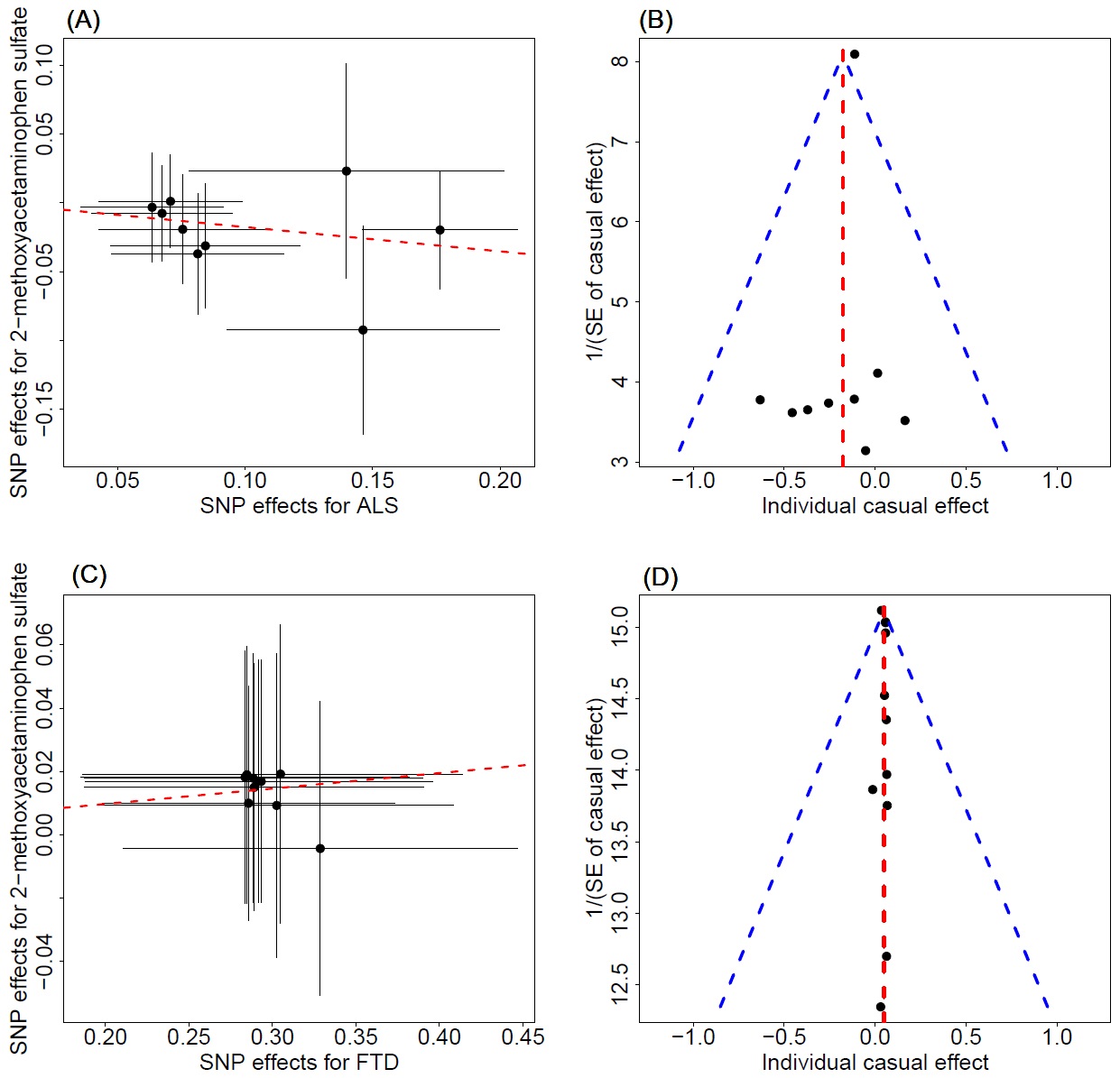


Figure S7. (A) Relationship between the SNP effect size estimates of ALS (x-axis) and the corresponding effect size estimates of 2-methoxyacetaminophen sulfate (y-axis). In the plot, the 95%CIs for the effect sizes of instruments on ALS are shown as horizontal lines, while the 95%CIs for the effect sizes of instruments on 2-methoxyacetaminophen sulfate are shown as vertical lines. The line in red represents the estimated causal effect of ALS on 2-methoxyacetaminophen sulfate with the IVW method; (B) Funnel plot for single causal effect estimates of ALS on 2-methoxyacetaminophen sulfate; the horizontal dot line denotes the overall estimated causal effect with IVW method; (C) Relationship between the SNP effect size estimates of FTD (x-axis) and the corresponding effect size estimates of 2-methoxyacetaminophen sulfate (y-axis). In the plot, the 95%CIs for the effect sizes of instruments on FTD are shown as horizontal lines, while the 95%CIs for the effect sizes of instruments on 2-methoxyacetaminophen sulfate are shown as vertical lines. The line in red represents the estimated causal effect of FTD on 2-methoxyacetaminophen sulfate with the IVW method; (D) Funnel plot for single causal effect estimates of FTD on 2-methoxyacetaminophen sulfate; the horizontal dot line denotes the overall estimated causal effect with IVW method.


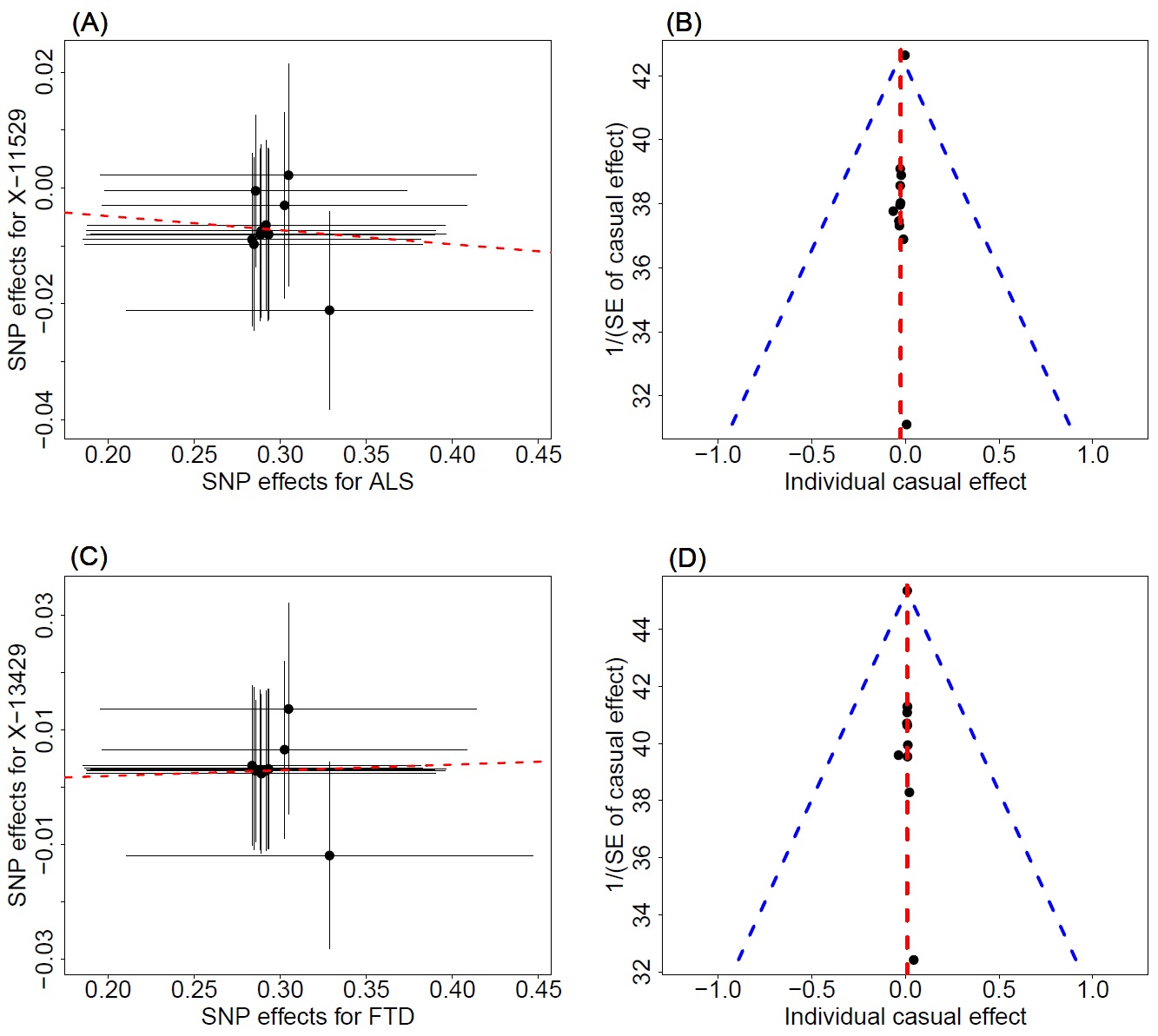


Figure S8. (A) Relationship between the SNP effect size estimates of FTD (x-axis) and the corresponding effect size estimates of X-11529 (y-axis). In the plot, the 95%CIs for the effect sizes of instruments on FTD are shown as horizontal lines, while the 95%CIs for the effect sizes of instruments on X-11529 are shown as vertical lines. The line in red represents the estimated causal effect of FTD on X-11529 with the IVW method; (B) Funnel plot for single causal effect estimates of FTD on X-11529; the horizontal dot line denotes the overall estimated causal effect with IVW method.; (C) Relationship between the SNP effect size estimates of FTD (x-axis) and the corresponding effect size estimates of X-13429 (y-axis). In the plot, the 95%CIs for the effect sizes of instruments on FTD are shown as horizontal lines, while the 95%CIs for the effect sizes of instruments on X-13429 are shown as vertical lines. The line in red represents the estimated causal effect of FTD on X-13429 with the IVW method; (D) Funnel plot for single causal effect estimates of FTD on X-13429; the horizontal dot line denotes the overall estimated causal effect with IVW method

References

1. Purcell S, Neale B, Todd-Brown K, Thomas L, Ferreira MA, Bender D, Maller J, Sklar P, de Bakker PI, Daly MJ *et al*: **PLINK: a tool set for whole-genome association and population-based linkage analyses**. *Am J Hum Genet* 2007, **81**(3):559-575.

2. The 1000 Genomes Project Consortium: **A global reference for human genetic variation**. *Nature* 2015, **526**(7571):68-74.

3. Zeng P, Zhou X: **Causal Association Between Birth Weight and Adult Diseases: Evidence From a Mendelian Randomization Analysis**. *Front Genet* 2019, **10**.

4. Larsson SC, Burgess S, Michaëlsson K: **Association of Genetic Variants Related to Serum Calcium Levels With Coronary Artery Disease and Myocardial Infarction**. *JAMA* 2017, **318**(4):371-380.

5. Ahmad OS, Morris JA, Mujammami M, Forgetta V, Leong A, Li R, Turgeon M, Greenwood CMT, Thanassoulis G, Meigs JB *et al*: **A Mendelian randomization study of the effect of type-2 diabetes on coronary heart disease**. *Nat Commun* 2015, **6**.

6. Giambartolomei C, Vukcevic D, Schadt EE, Franke L, Hingorani AD, Wallace C, Plagnol V: **Bayesian test for colocalisation between pairs of genetic association studies using summary statistics**. *PLoS genetics* 2014, **10**(5):e1004383.
